# Category fluency and creative potential in semantic aphasia

**DOI:** 10.1111/jnp.70019

**Published:** 2025-12-10

**Authors:** Hannah E. Thompson, Paul T. Sowden, Lucy Cogdell‐Brooke, Ines R. Violante, Beth Jefferies

**Affiliations:** ^1^ Department of Psychology and Neuroscience City St George's, University of London London UK; ^2^ School of Psychology University of Surrey Guildford UK; ^3^ School of Psychology and Social Sciences University of Winchester Winchester UK; ^4^ School of Biomedical Engineering & Imaging Sciences King's College London London UK; ^5^ Department of Psychology University of York York UK

**Keywords:** category fluency, creative potential, creativity, semantic aphasia

## Abstract

Creative cognition involves linking weakly or unrelated concepts, enabled by semantic control (inhibiting dominant associations to retrieve weaker ones) or through spreading activation within the semantic system. Semantic aphasia (SA) patients have impaired semantic control despite relatively preserved semantic representations. To date, no studies have examined creativity in SA. It remains unclear how impaired control affects patients' creative potential, and whether spreading activation alone supports this. Creative potential was assessed across three experiments. Experiments 1 and 2 involved 11 SA patients and 25 controls; Experiment 3 included 13 SA patients and 14 controls. In Experiment 1 (category judgement), participants selected five targets from distractors across 24 categories with differing coherence levels (shared features among members). Experiment 2 (constrained category fluency) involved generating five exemplars per category. Creative potential was measured via uniqueness, flexibility, semantic distance and creativity ratings. Experiment 3 (unconstrained fluency) asked participants to name as many Animals as possible in 1 minute, with additional measures of clustering and switching. Although SA cases were unable to shape retrieval to pre‐defined associations (in the category judgement task), they showed creative potential in the constrained fluency task. In the unconstrained fluency task, patients were less able to use strategies. However, with fluency controlled, no group differences in creative potential existed. These findings provide the first neuropsychological evidence that spreading activation, even with impaired semantic control, can support creative responses. Creative potential in SA depends on task demands, aligning with broader findings of patients' sensitivity to context.

## INTRODUCTION

Semantic cognition refers to our capacity to use acquired concepts about the world (Lambon Ralph et al., 2017). Semantic cognition has at least two components. Firstly, an automatic mechanism allows for spreading activation amongst highly related conceptual representations. Secondly, semantic control mechanisms allow for the flexible manipulation of conceptual knowledge in line with the current context (Jefferies, 2013). Semantic Aphasia (SA) patients have disrupted semantic control, with relatively spared semantic representations (Jefferies & Lambon Ralph, 2006; Noonan et al., 2010). This means that SA cases have difficulty overcoming dominant responses in order to retrieve weaker, yet task‐relevant, concepts (Thompson et al., 2022). Patients can accurately match two highly related stimuli such as SALT and PEPPER but show impairments when the task requires internally generated constraints, such as matching weak associations (e.g. SALT and SUGAR, Noonan et al., 2010). Further, along with often pronounced language output deficits, these patients show parallel non‐verbal semantic retrieval impairments in picture comprehension and object use (Corbett et al., 2009; Jefferies & Lambon Ralph, 2006).

### Creative potential in semantic aphasia

Semantic cognition, including conceptual representation and semantic control, plays a pivotal role in creativity. More creative responses are distinguished by the ability to make connections between seemingly unrelated, or remotely related, concepts (Beaty & Kenett, 2023; Mednick, 1962; Sowden et al., 2015). Importantly, creativity and creative potential are distinct. Creative potential reflects what is possible, rather than what someone has done (Lubart et al., 2013, 2022). Therefore, even in tasks which do not instruct someone to ‘be creative’, creative potential can be explored. This has been widely exploited in work using category fluency and free association tasks to explore how the structure and function of semantic networks, their creative potential, can predict creativity (Kenett et al., 2014). SA patients, with impaired goal‐directed cognition (Thompson et al., 2018), are likely to find tasks with the instructions to ‘be creative’ problematic, because it triggers top‐down processing (e.g. Nusbaum et al., 2014).

A key model of creativity, the associative model of creativity (Beaty & Kenett, 2023), suggests two routes to creative responses. First, spreading activation within the semantic memory network structure, such that in more interconnected networks, more remote concepts are accessed through spreading activation, reflecting greater creative potential. Second, semantic control can intentionally inhibit dominant responses to reach more weakly connected concepts.

Semantic models have long argued that spreading activation between connected responses occurs, with semantic distance affecting response time (Collins & Loftus, 1975). This is shown in speeded responding to related concepts (e.g. Neely, 1977). Within categories, more typical items for a category are easier to access than less typical ones (Rosch, 1975). This suggests that activation spreads more efficiently through densely connected nodes and reaches prototypical exemplars and close associates more rapidly. Semantic networks and creativity are therefore intimately related individuals with more richly interconnected semantic networks show enhanced fluency and flexibility (Kenett et al., 2014).

Prevailing models of creativity are consistent with the view that creative thought emerges from the interaction of two complementary components: an associative process and an analytical process (see Sowden et al., 2015). We focus particularly on the associative model of creativity because it integrates some of the neuroscientific evidence with traditional models of creativity and so offers a mechanistic explanation for routes to creativity. Unlike attentional accounts emphasizing gating internal and external information (Zabelina et al., 2016), or individual differences perspectives, including personality, motivation and mood (Amabile & Pratt, 2016; Baas et al., 2008; Feist, 2019), the associative model is well suited to our context, where the tasks rely on the semantic system and patients show selected controlled retrieval impairments.

We expect that SA patients will be more reliant on forming associations through spreading activation, because their ability to access task‐relevant concepts through semantic control is disrupted. This may limit their potential for creativity. Indeed, neuropsychological studies have shown impairments of creativity in executively impaired patients (de Souza et al., 2010; Rankin et al., 2007; Shamay‐Tsoory et al., 2011). However, there are also explorations of counterintuitive de novo *increases* in creativity. For example, neurodegeneration following frontotemporal dementia was shown to increase artistic production and ability (de Souza et al., 2014; Miller et al., 1996, 1998; Miller & Miller, 2013; Wu et al., 2015). Similar reports have been made about individuals with other neurodegenerative conditions such as prefrontal damage (Shamay‐Tsoory et al., 2011). This has been interpreted as a disinhibited or disrupted system (Chan et al., 2023). Creativity has not been explored in SA. The present research addresses this gap by exploring what we can learn about the relationship between disrupted semantic control and potential for creativity in SA patients, as revealed through category fluency and category judgment tasks.

### Category fluency and creative potential

Category fluency tasks assess the ability to generate items from a particular semantic category (e.g. Animals), usually within a given time frame (Henderson et al., 2023; Runco, 2010). Category fluency tasks have been used to construct and characterise semantic networks, and hence potential for spreading activation between both close and distant exemplars, linking to a core aspect of creative potential, divergent thinking (Beaty et al., 2014; Christensen & Kenett, 2023; Kenett, 2019). Performance on category fluency tasks is governed by spreading semantic activation to activate relevant concepts, alongside control to guide retrieval to relevant but less dominant exemplars (Demetriou & Holtzer, 2017; Fernaeus & Almkvist, 1998; Hurks et al., 2006; Schwartz et al., 2003). This broad semantic retrieval ability predicts creative cognition (Gerver et al., 2023).

SA patients produce fewer items on category fluency tasks than semantic dementia patients (Rogers et al., 2015). There are several reasons why deficits may be found. Firstly, patients commonly have deficits in both expressive and receptive semantic tasks, but expressive tasks typically place additional strain on semantic control, as they usually provide less context to guide retrieval (Noonan et al., 2013). Secondly, fluency tasks require *multiple* responses, demanding significant semantic control to select among competitors, followed by inhibiting this response and selecting a different, equally accurate answer, all the while inhibiting other related but less relevant responses (Rogers et al., 2015). SA patients have been previously shown to be particularly sensitive to repeated stimuli and time‐pressured demands on the semantic‐executive system, with reduced performance over time (Gardner et al., 2012; Thompson et al., 2015). Finally, broader executive strategies may be essential to generating multiple responses (Troyer et al., 1997), and this strategy use may be impaired in SA. Fluency impairments have been shown to reflect broader executive control deficits (Bose et al., 2022). In healthy controls, clustering within subcategories before switching to another subcategory cluster is commonly found in fluency tasks, bolstering the overall number of items retrieved (Murphy et al., 2006; Weakley & Schmitter‐Edgecombe, 2014). This switching between subcategories is thought to have an executive component (Abwender et al., 2001; Hirshorn & Thompson‐Schill, 2006; Raboutet et al., 2010).

If the task demands of category fluency tasks are reduced, for example, by limiting the number of responses needed or reducing the time constraint, spreading activation alone may be sufficient for creative potential in patients' responses. In this context, some evidence suggests that damage to semantic and control structures may even ‘free’ associations from the limitations of conventional thought (Amer et al., 2016; Reverberi et al., 2005; Shamay‐Tsoory et al., 2011), in which case SA patients might show more diverse response patterns. However, we anticipate that controls will structure their responses to progressively explore semantic space in a way that SA patients do not, because this requires both control and strategic intervention (Troyer et al., 1997). To explore whether spreading activation is sufficient for creative potential, we analyze responses given by patients and controls on two versions of a generative category fluency task, one only requiring a limited set of responses thereby emphasizing spreading activation, and the other an unconstrained version requiring the production of multiple exemplars and expected to require semantic control. We predict that SA patients will show relatively equivalent creative potential to controls on the reduced response version of the category fluency task, and when fluency is controlled for in the unconstrained version.

### Category coherence

Categories used in a category fluency task can be wide‐ranging (Barsalou, 1983). For example, an item such as bread can occur in multiple categories, such as things in a bakery and things that are brown. These categories are fundamentally different in terms of their coherence, that is, the relationship between the items within them (Murphy & Medin, 1985). Categories like things that are brown have very few aspects in common (showing low coherence), compared to those where members have many aspects in common (high coherence, Davey et al., 2016). For highly coherent concepts, dominant features can be easily retrieved (Jackson et al., 2016; Lau et al., 2013). However, for low coherence categories, control may be required to select items based on a specific feature in the absence of a global relationship (Badre et al., 2005; Davey et al., 2016).

Therefore, an additional aim of this study is to assess the impact of category coherence on creative potential. We will compare performance on low and high coherence categories in a generative fluency task, alongside a category judgement task—selecting target category members amongst related but irrelevant distractors. We anticipate performance to be impaired on category judgement, most notably for low coherence categories. Conversely, for the generative fluency task, we anticipate that greater freedom in acceptable responses for low coherence categories—due to category boundaries being less clearly defined—may benefit patients and negate any difficulty with the lack of coherence between items.

### Modality

Category fluency tasks are often explored in the verbal domain. There is some debate as to whether category fluency is a language or executive task (Whiteside et al., 2016), although there is evidence that it may relate more to language capabilities (Henry et al., 2015). Semantic aphasia transcends standard aphasia classifications, with patients classified only based on multimodal comprehension impairments (Jefferies & Lambon Ralph, 2006). Therefore, those with SA may have fluent or non‐fluent profiles, but equivalent verbal and non‐verbal deficits (Corbett et al., 2009; Gardner et al., 2012; Jefferies & Lambon Ralph, 2006), due to disruption of multimodal semantic control processes. Therefore, a final aim of this study is directly comparing performance on the same tasks across modalities. It is predicted that there will be parallel performance in verbal and non‐verbal tasks, in line with previous work in SA.

### Summary and predictions

In summary, the associative model of creativity asserts that creative responding is underpinned by the formation of connections between more remotely associated items. These associations result from spreading activation and semantic control. SA patients have disrupted semantic control mechanisms, with an intact semantic store. We predict that SA patients will have normal creative potential in tasks which have reduced task demands, and when controlling for fluency. This is due to intact spreading activation. We predict that these patterns will be equivalent across modalities and for low and high coherence categories. However, we predict that reduced strategy use, such as switching between subcategories, will be evident. We further predict that SA patients will show impaired performance on a matched category judgment task, where items need to be inhibited and competitively selected, particularly for low coherence categories, which place the highest demands on semantic control.

## MATERIALS AND METHODS

### Participants

SA patients were compared with healthy, age‐matched controls. All participants were native English speakers. Participants' consent was obtained according to the Declaration of Helsinki and approval was provided by the relevant Local Ethics Committee in each case.

#### SA patients

A neuropsychological screening test was conducted on stroke patients recruited via community groups in Surrey, West Sussex and York. Background neuropsychological testing (Table S1) was used to classify patients as having Semantic Aphasia (SA) according to previously published guidelines (Jefferies & Lambon Ralph, 2006), that is, patients were selected to show impaired semantic cognition in both verbal and non‐verbal tasks.

A total of 16 Semantic Aphasia (SA) cases with a left‐hemisphere cerebrovascular accident (CVA) took part across three experiments. Of our 16 SA patients, there were nine males, with an average age of 62, and an average age leaving education of 18 (see Table S1). All patients had chronic impairments from a CVA at least a year before the study. There were 11 cases in Experiments 1 and 2, and 13 were in Experiment 3 (8 cases took part in all 3 experiments, alongside 5 additional cases in Experiment 3, and 3 additional cases in Experiments 1 and 2). There were no statistical differences between those who did and did not take part in all experiments in demographics (*t*(14) ≤ 1.528, *p* ≥ .149) or background tasks (*t*(11–14) ≤ 2.021, *p* ≥ .064). Fluency was only measured for those able to produce speech; patients unable to produce speech did not take part in Experiment 3, or verbal subsections of Experiment 2 (see below for further details).

#### Controls

For Experiments 1 and 2, 25 controls took part (eight males), with an average age of 63, and an average age leaving education of 20. For Experiment 3, there were 14 controls (six males), with an average age of 67, and an average age leaving education of 20. Controls were recruited from the general population, via advertisement and opportunity sampling, and matched patients in age and education (*t* < 1). All controls had no history of neurological disease or damage and scored at least 28/30 in the Mini Mental State Exam (Folstein et al., 1983).

### Neuropsychological assessment

Patients completed a range of background neuropsychological tasks, interspersed with our main experimental tasks. It was a requirement of their SA classification to show deficits in semantic cognition across modalities. There were multiple testing sessions, each session lasting ~1 h.

#### Semantic and linguistic tasks

Semantic tasks included: Four shortened tasks from the Cambridge Semantic Battery (Bozeat et al., 2000), which comprised (i, ii) 25 items each from the associative word and picture Camel and Cactus test (CCT), (iii) 16 items from the spoken word‐to‐picture matching (WPM) and (iv) 16 items from the picture naming tasks. Other tasks included (v) a 96‐item synonym judgment (Jefferies et al., 2009) which manipulated imageability and frequency, (vi) a 30‐item ambiguity task (Noonan et al., 2010), which used polysemous words with dominant or subordinate relations (e.g. Leaf and Tree or Leaf and Page) and (vii) a 37‐item alternative object use task (Corbett et al., 2011), selecting canonical or non‐canonical objects to use to perform an action (e.g. ‘kill a fly’ with Flyswat or Newspaper). Language tasks included the cookie theft task (Goodglass et al., 2001), recording words produced per minute and word repetition (from PALPA 9, Kay et al., 1996), including high and low imageability words.

#### Non‐semantic tasks

Non‐semantic tasks included: three tasks from the WMS‐IV (Wechsler, 2009): (i) The Brief Cognitive Status Exam (BCSE), (ii) forwards digit span and (iii) symbol span. Other tasks included (iv) Raven's Coloured Progressive Matrices parts A, AB and B (RCPM, Raven, 1962), (v) Trail Making (parts A and B, Reitan, 1958) and (vi) the Brixton Spatial Anticipation Test (BSAT, Burgess & Shallice, 1997).

#### Neuropsychological scores

Background data for patients are presented in Table S1. All patients showed impairments in verbal and non‐verbal semantic tasks, required for the classification of SA (Jefferies & Lambon Ralph, 2006). There was variable performance in tasks that tap other linguistic processes, such as naming, fluency and repetition, as would be expected from the selection criteria, which classify only according to comprehension.

### Neuroimaging data

**FIGURE 1:**
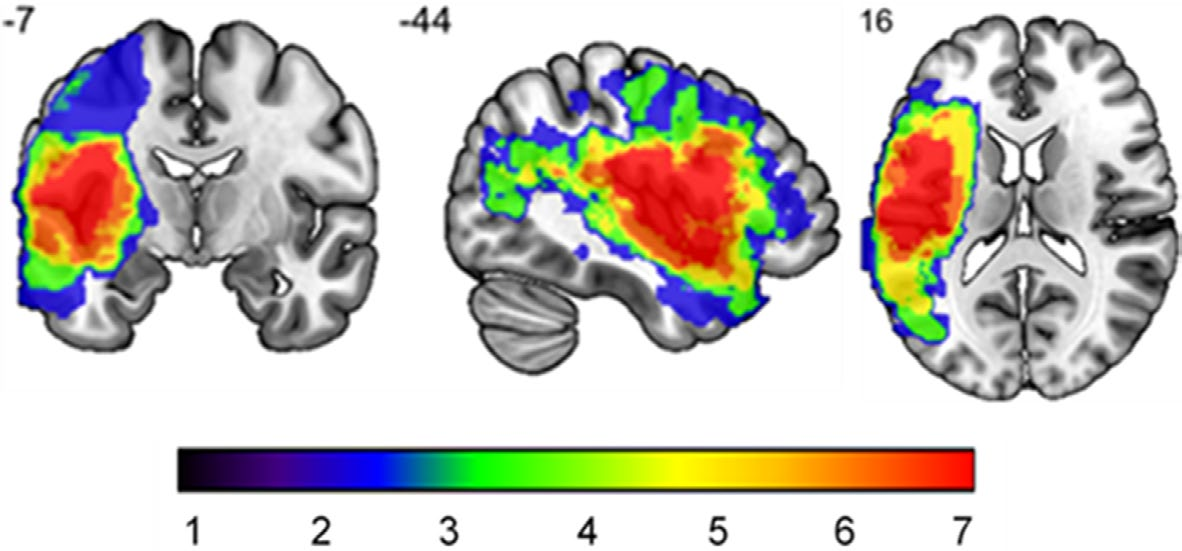
Lesion map of a sample of seven SA patients included in the study. The colour bar shows the amount of patient overlap.

Scans were available for seven patients and were used to check whether areas of damage related to semantic control regions. The analysis method is presented in Supplementary Analysis S1. The lesion overlap for the patient group is shown in Figure 1.

### Analytic approaches

Our standard analytic approach was to use analysis of variance with post‐hoc analysis via *t*‐tests where applicable, run with the False Discovery Rate controlled at 5% using the Benjamini‐Hochberg procedure (Benjamini & Hochberg, 1995). Where non‐significant main effects of group were observed, we supplemented inferential statistics with Bayesian analyses to check for evidence of non‐significance (Dienes, 2014). These used default priors. Robustness checks were also performed using a range of prior specifications. Deviations from our standard analytic approach are described where they occur.

Where creative potential was explored, we measured different indices, in line with previous work (e.g. Plucker et al., 2011; Reiter‐Palmon et al., 2019; Silvia et al., 2008). For Experiment 2, we explored (1) uniqueness (number of responses which were only produced by one individual), (2) flexibility between responses, measured using Latent Semantic Analysis (LSA, Beaty et al., 2014) and (3) subjective ratings of creativity. For Experiment 3, we could additionally explore flexibility between responses using switching and clustering measurements and forward flow (the average semantic distance of a response from all previous responses, Gray et al., 2019). Forward flow and LSA offer complementary ways to explore semantic distance travelled, with forward flow suitable for tasks with more responses, such as the unrestricted fluency task. Forward flow helps test our prediction that controls will structure their responses to progressively explore semantic space enabling them to ‘travel’ further in a semantic space than SA patients.

## EXPERIMENT 1: CATEGORY JUDGEMENT TASK

### Method

#### Participants

Data were available for all 11 SA cases. Across 25 controls, there were 12/100 data points missing (from coherence and modality manipulations), due to testing limitations, for example, not all controls took part in every available session.

#### Stimuli

Twenty‐four categories, 13 high coherence and 11 low coherence, were used from Lupyan et al. (2012). These categories were tested in both category fluency and category judgment tasks and across verbal and non‐verbal modalities (see Table 1). Full details of categories, targets and distractors used in the category judgment task can be seen in Table S2.

**TABLE 1:** Categories used for category fluency and judgment tasks.

	High coherence	Low coherence
Categories	Farm animals	Things that are round
	Items of clothing on top half	Things made of wood
	Flying animals	Things that are small
	Transport	Things that are large
	Animals that live in water	Things that are soft
	Exotic fruits	Things that have handles
	Facial features	Things that are thin
	Insects	Things with stripes
	Vegetables	Things that make a noise
	Kitchen appliances	Things that are square
	Dangerous animals	Things that are move on wheels
	Objects that hold water	
	Zoo animals	

#### Procedure

All of our experimental tasks were run on separate testing sessions. For each category, participants were presented with 20 items and instructed to select the five items that fitted the category the best (e.g. Farm animals). Five items were targets (e.g. Cow, Sheep, Pig, Horse, Chicken) and 15 were distractors. There were three distractor types, although the variety was not associated with specific predictions about group differences: categorical (e.g. Camel), associative (e.g. Barn) and unrelated (e.g. Pineapple). Categorical distractors were only used in the high coherence categories, as it was not possible for low coherence categories. Items appeared as a target only once; however, some unrelated distractors were reused where necessary, but not more than twice. The non‐verbal task used line drawings from Snodgrass and Vanderwart (1980), supplemented where necessary. The verbal task used written words, which were read aloud to patients where required. Category order was counterbalanced between participants.

### Results

**FIGURE 2:**
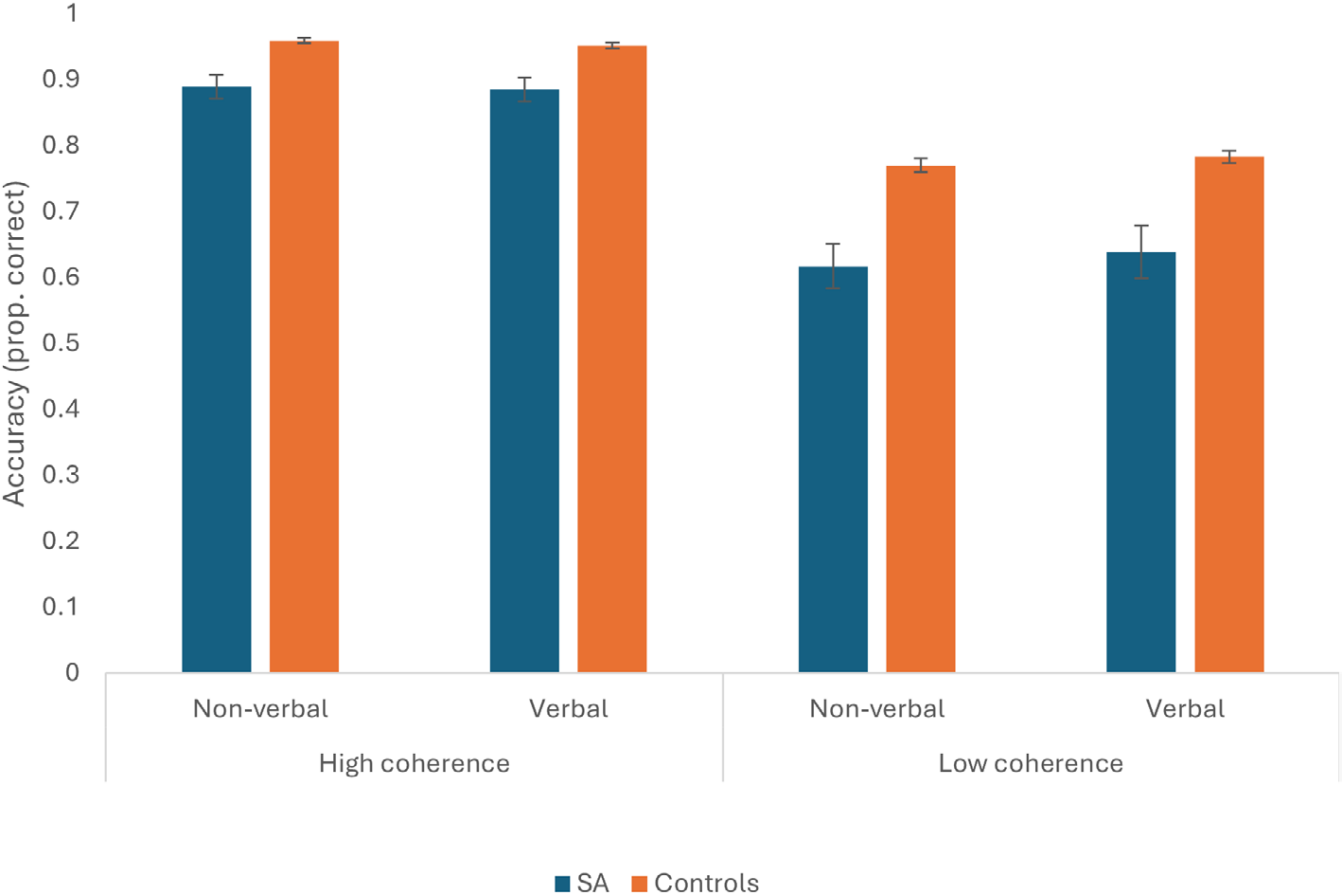
Proportion correct for category judgment task across modalities, for SA patients and controls across high and low coherence categories. Error bars show the standard error of the mean.

There was a significant main effect of group, with accuracy lower for patients than controls overall: *F*(1,27) = 32.610, *p* < .001, *η*_p_^2^ = .547; shown in Figure 2. There was also a significant main effect of category coherence, with lower accuracy for low coherence categories: *F*(1,27) = 270.754, *p* < .001, *η*_p_^2^ = .909 and an interaction between category coherence and group: *F*(1,27) = 8.061, *p* = .008, *η*_p_^2^ = .230. Modality showed no main effect (*F* < 1), and the interaction with group was not significant (*F* < 1). The coherence and modality interaction did not reach significance: *F*(1,27) = 3.690, *p* = .065, *η*_p_^2^ = .120, and neither did the three‐way interaction (*F* < 1).

To explore the coherence by group interaction further, the difference between high and low coherence was computed, averaging across modalities. SA cases showed a larger coherence difference than controls: *t*(27) = 2.839, *p* = .008, *d* = 1.087, reflecting difficulties internally shaping semantic retrieval when external constraints are reduced. Error types are explored in Supplementary Analysis S2.

### Interim discussion

In line with previous work (Jefferies & Lambon Ralph, 2006; Noonan et al., 2010; Thompson et al., 2022), patients show impaired accuracy where they are required to select a pre‐identified target among related distractors across modalities—a task which requires semantic control. Accuracy was notably reduced for low coherence categories. Targets in these categories share few associations, offering less support during retrieval. High coherence categories benefit from denser semantic links to reduce competition from distractors—where low coherence categories are more vulnerable to interference. Next, we aimed to explore whether patients could show creative potential in a *generative* task with reduced control demands.

## EXPERIMENT 2: RESTRICTED CATEGORY FLUENCY TASK

### Method

#### Participants

Out of our overall sample of 11 patients and 25 control participants, some data points were missing due to testing constraints, data availability and participants being unable to complete certain tasks (e.g. non‐verbal patients). As a result, the number of participants included in each condition varied slightly: (1) verbal high convergence: 20 controls, 8 patients; (2) verbal low convergence: 19 controls, 8 patients; (3) non‐verbal high convergence: 24 controls, 11 patients and (4) non‐verbal low convergence: 23 controls 11 patients.

#### Stimuli and procedure

The same categories from the judgement task were used in this task—the category was always available to refer to in written form as well as read aloud. Participants were asked to generate five responses per condition, and there was no time limit for completing the task. In the non‐verbal task, participants were given a sheet with five boxes and could draw five responses. In the verbal task, participants provided oral responses or had the option of writing their responses. Note that Experiment 1 involved evaluating the relative typicality within a category (e.g. picking five items among distractors, for example, ‘pineapple’ over ‘cherry’ for Exotic fruits), whereas Experiment 2 was a generative task. Because of this, it is possible that a response was incorrect on Experiment 1, but acceptable on Experiment 2, if 90% of independent raters judged it as appropriate for the category.

### Results

**FIGURE 3:**
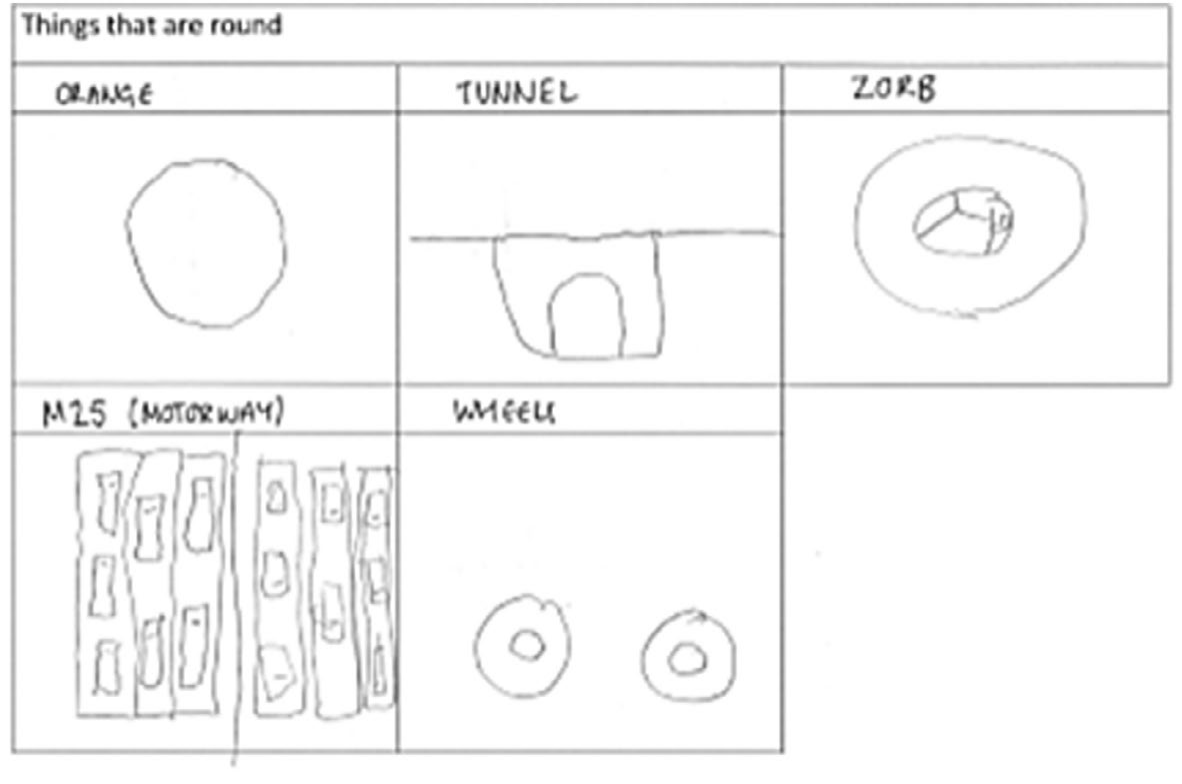
Example patient response to non‐verbal restricted category fluency task.

Figure 3 shows a typical example of creative responses found in the patient sample for the category ‘things that are round’. Invalid response analysis is shown in Supplementary Analysis S3.

#### Uniqueness

**FIGURE 4:**
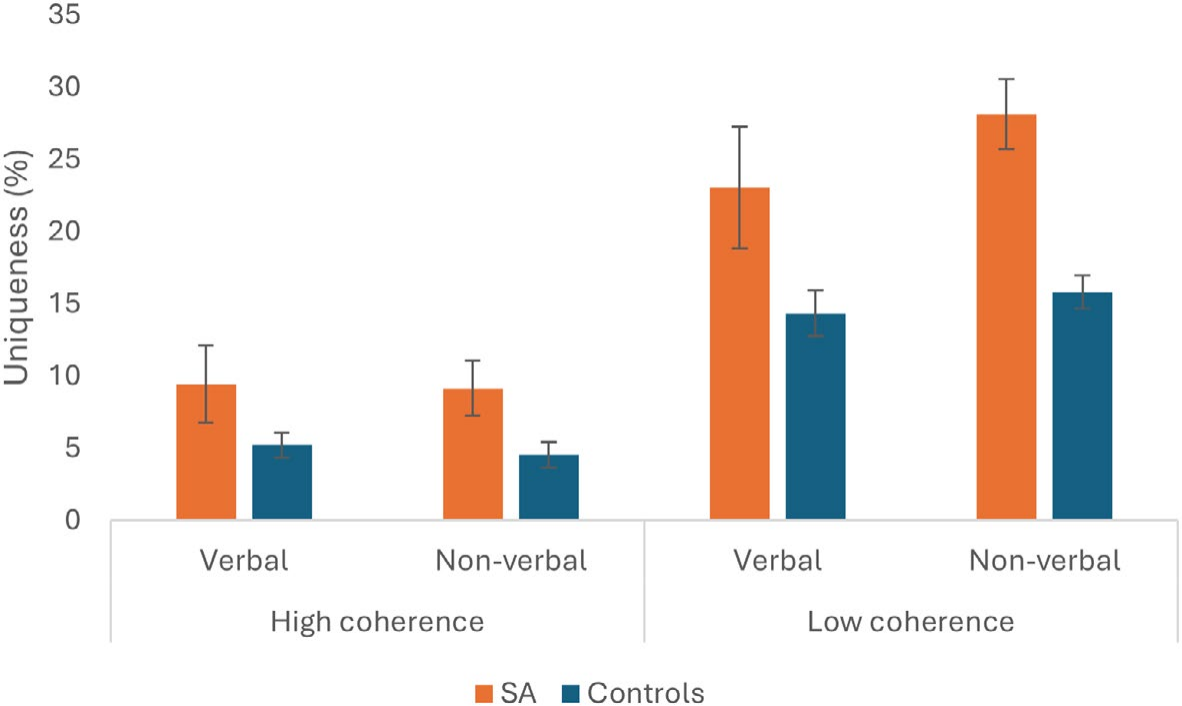
Percentage of unique responses produced by patients and controls across conditions in the restricted category fluency task. Error bars show standard error of mean.

Uniqueness is a key index of creative potential. After removing any invalid responses, responses produced by one person were given a score of 1, all other responses scored 0. The number of unique responses was scored as a percentage of total responses per category. SA patients were more likely to produce unique responses than controls: *F*(1,21) = 12.217, *p* = .002, *η*_p_^2^ = .368 (see Figure 4). There were more unique responses in the low coherence than high coherence categories: *F*(1,21) = 117.269, *p* < .001, *η*_p_^2^ = .848. There was a significant interaction of coherence and group: *F*(1,21) = 5.137, *p* = .034, *η*_p_^2^ = .197.

SA cases produced more unique responses for the low coherence categories. When averaging across modalities, the group difference was less pronounced for high coherence: *t*(26) = 2.355, *p* = .026, *d* = .985, than low coherence: *t*(25) = 4.251, *p* < .001, *d* = 1.791. The effect of modality was not significant: *F* < 1, and there was no interaction with group: *F* < 1. Coherence and modality interacted: *F*(1,21) = 8.135, *p* = .010, *η*_p_^2^ = .279, reflecting a larger effect size of coherence for non‐verbal categories: *t*(32) = 11.537, *p* < .001, *d* = 2.008, than verbal categories: *t*(24) = 5.526, *p* < .001, *d* = 1.105. There was no three‐way interaction: *F* < 1, this effect was the same across groups.

#### Flexibility

A second index of creative potential is flexibility between responses. As our task was limited to five responses, we used Latent Semantic Analysis (LSA, Beaty et al., 2014; http://wordvec.colorado.edu/index.html) as a proxy measure. More flexibility would be reflected in lower associations, suggestive of more diverse responses and higher creative potential.

**FIGURE 5:**
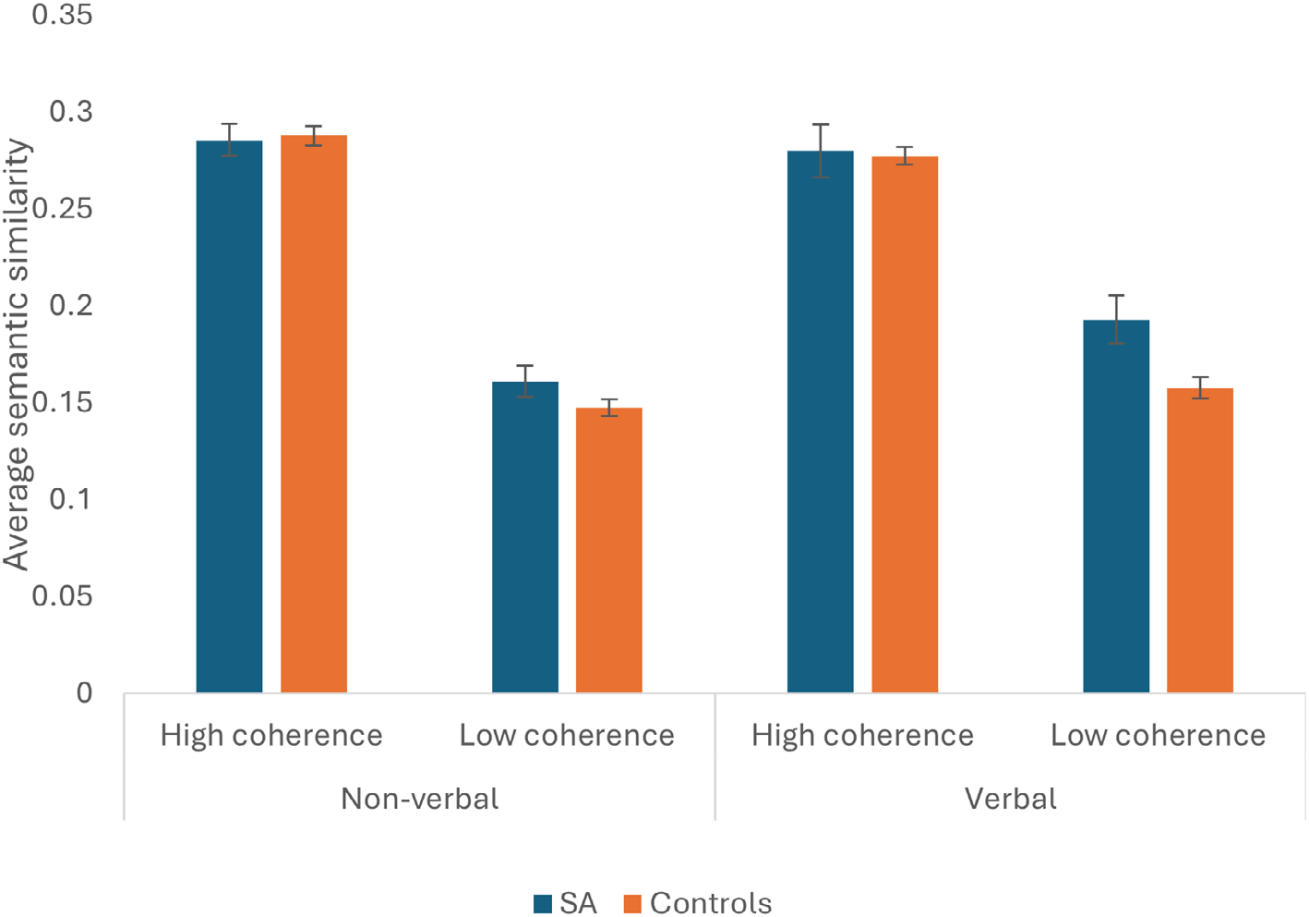
Average similarity scores (max. possible = 1) between responses given for each modality and level of category coherence for patients and controls. Error bars show standard error of mean.

There was a significant main effect of group, with higher scores from patients, reflecting greater similarity of responses: *F*(1,20) = 7.06, *p* = .015, *η*_p_^2^ = .261. There was a main effect of category coherence with both groups producing more related responses to high coherence categories: *F*(1,20) = 600.82, *p* < .001, *η*_p_^2^ = .968. The effect of modality was not significant: *F*(1,20) = 3.491, *p* = .076, *η*_p_^2^ = .149. There was an interaction between modality and category coherence: *F*(1,20) = 5.54, *p* = .029, *η*_p_^2^ = .217. There was greater semantic similarity between responses to the verbal compared with non‐verbal low coherence categories: *t*(23) = 2.789, *p* = .010, *d* = .569, but there was no modality difference for high coherence categories (*t* < 1). Other interactions did not reach significance: *F* ≤ 3.525, *p* ≥ .075. This is shown in Figure 5. An analysis of the similarity of responses to the category prompt is shown in Supplementary Analysis S4.

#### Creativity ratings

We also used rater‐based scoring to assess the ‘creativity’ of participants' responses, acknowledging that participants were not explicitly instructed to ‘be creative’ (Wei et al., 2024). Raters (see Uniqueness for demographic information) were asked to consider whether each response was creative for the category, with a creative response being ‘…considered in its novelty, usefulness and surprise’. There was an 8‐point scale (0–7), zero as a valid but not creative response to seven as extremely creative. Scores were averaged across raters. Ratings can be seen in Table S3.

Patients were as creative as controls as measured by ratings: *F*(1,19) = 1.599, *p* = .221, *η*_p_^2^ = .078, BF_incl_ = .62. There was higher creativity in low compared to high coherence categories: *F*(1,19) = 171.235, *p* < .001, *η*_p_^2^ = .900, which did not interact with group (*F*(1,19) = 2.986, *p* = .100, *η*_p_^2^ = .136), although Bayesian analysis suggested that models containing the group × coherence interaction were slightly more likely (BF_incl_ = 1.33). Creativity was higher for non‐verbal than verbal tasks: *F*(1,19) = 8.726, *p* = .008, *η*_p_^2^ = .315, which did not interact with group: *F* < 1. Coherence and modality did not interact: *F*(1,19) = 1.84, *p* = .191, *η*_p_^2^ = .088, and there was no three‐way interaction: *F*(1,19) = 1.960, *p* = .178, *η*_p_^2^ = .094. This is shown in Figure S1.

#### Typicality

Responses may be considered more creative if they are low frequency for the category (e.g. Rambutan for Exotic fruits); however, they may also be creative if they push the boundaries of what may be typical for the category (e.g., avocado for Exotic fruits). To explore the patients' creative potential further, the typicality of the responses was explored. There was a minimum of 24 raters per item (24–30 raters, average age = 20, min = 18, max = 25, SD = 1.6). This was for how typical the response was, on a scale of 0 = does not fit the category, 1 = very untypical, and 7 = very typical for the category. Excluding invalid responses, there was a main effect of group: *F*(1,68) = 29.36, *p* < .001, partial *η* ^2^ = .30, with less typical responses from patients compared to controls. There was also a significant main effect of coherence: *F*(1,68) = 234.40, *p* < .001, partial *η* ^2^ = .80, with low coherence categories rated less typical than high coherence categories. The interaction was not significant: *F* < 1.

### Interim discussion

In the restricted category fluency task, with reduced task demands and maximal flexibility in self‐generation, patients show significantly more unique responses than controls, particularly for low coherence categories, and were deemed creative according to ratings. However, their responses were also more likely to be invalid and less typical for the category. Thus, patients may have achieved creative potential through pushing the boundaries of what is acceptable for the category (e.g. ‘avocado’ for Exotic fruit or ‘mouse’ for Farm animals).

## EXPERIMENT 3: UNRESTRICTED CATEGORY FLUENCY

### Method

#### Procedure

The unrestricted category fluency task was conducted by participants being given a category and 60 s to verbalize as many responses as possible. A category that has clear subcategories was selected (Animals). We could therefore look beyond the total fluency to explore clustering and switching, alongside creativity and uniqueness. Responses were recorded in 15‐s interval boxes, giving us four time points across the task. Time is important to explore, as creativity has been shown to increase across time (Beaty & Silvia, 2012), where closer or more typical responses are arrived at earlier, and more remote associations come later (Mednick, 1962).

#### Analytical method

In Analysis 1, we looked at total responses, exploring fluency across time, number of switches and cluster size. In Analysis two, we controlled for fluency and explored creativity and uniqueness. As in Experiment 2, a set of raters (see Creativity) was asked to identify invalid responses for exclusion from further analysis. There were no invalid responses in this experiment.

### Results

Raw data are shown in Table 2.

**TABLE 2:** Data showing responses across time for patients and controls, as well as the total and/or average where appropriate.

	Patients						Controls					
	0–15	15–30	30–45	45–60	Total	Average	0–15	15–30	30–45	45–60	Total	Average
Fluency	3.46 (1.5)	2.08 (2.0)	1.54 (1.3)	.77 (1.0)	7.85 (4.1)	1.96 (1.0)	9.29 (1.3)	6.79 (3.0)	5.57 (1.9)	4.50 (2.1)	26.14 (4.1)	6.54 (1.0)
Switching	.85 (.9)	1.46 (1.3)	.38 (.7)	.31 (.48)	3.00 (2.3)	.75 (.6)	3.14 (1.6)	2.43 (1.7)	2.00 (1.3)	1.71 (1.0)	9.29 (2.6)	2.32 (.7)
Creativity	1.42 (.4)	2.12 (1.4)	1.97 (1.4)	1.00 (1.2)	6.51 (2.3)	1.63 (2.3)	2.26 (.5)	2.58 (.5)	2.70 (.6)	2.46 (.9)	10.0 (1.9)	2.50 (.5)
Relative uniqueness	.42 (.2)	.82 (.1)	.72 (.2)	.45 (.2)		.60 (.1)	.65 (.1)	.81 (.1)	.85 (.1)	.79 (.1)		.78 (.5)
Unique %	.00 (0)	18.21 (30.0)	25.00 (38.2)	.00 (0)		10.80 (15.9)	7.53 (10.4)	18.71 (18.1)	20.16 (15.5)	23.17 (19.0)		17.4 (11.1)
Forward flow						.68 (.1)						.78 (.0)
Cluster size						2.07 (.6)						2.67 (.7)

*Note*: Mean (SD).

#### Analysis 1: The impact of time on performance

##### Overall fluency

There was a significant effect of time, with fluency declining over time: *F*(3,75) = 21.446, *p* < .001, *η*_p_^2^ = .462. There was a significant effect of group, with controls more fluent: *F*(1,25) = 133.240, *p* < .001, *η*_p_^2^ = .842. The interaction of time and group was not significant: *F*(3,75) = 1.815, *p* = .152, *η*_p_^2^ = .068. The linear trend was significant for time: *F*(1,25) = 99.328, *p* < .001, *η*_p_^2^ = .799.

##### Switching and clustering

We defined a category switch as a clear shift between distinct subcategories (e.g. salmon–cow). Importantly, we acknowledge that items can belong to multiple subcategories (e.g. a penguin may belong with Birds or arctic Animals, and a koala bear may belong with Bears or Australian animals). Our approach was similar to Troyer et al. (1997), who also allowed items to be assigned to multiple subcategories (e.g. tiger → Zoo, Feline). However, we adopted a more conservative approach—a ‘switch’ was only recorded when there was a clear boundary between subcategories, not where there was gradual thematic progression (e.g. rat–hedgehog–hare–deer). 10% of switch ratings were conducted by two experimenters, with 85.5% consistency, meaning 90% of switches were coded by a primary rater, with a subset double‐coded. Switches also gave the size of the cluster. However, clustering could not be explored by time block, as clusters often extended across block boundaries.

The average cluster size was larger for controls than for patients: *t*(25) = 2.420, *p* = .023, *d* = .932. Switching significantly reduced over time: *F*(3,75) = 4.949, *p* = .003, *η*_p_^2^ = .165. This was confirmed by a significant linear reduction in switching across time points: *F*(1, 25) = 14.700, *p* < .001, *η*_p_^2^ = .370. There was also a main effect of group, such that controls switched more than patients: *F*(1,25) = 44.136, *p* < .001, *η*_p_^2^ = .638. However, there was no time by group interaction: *F*(3,75) = 1.476, *p* = .228, *η*_p_^2^ = .056.

##### Forward flow

Forward flow calculates the average semantic distance of a response from all previous responses and is related to creativity (Gray et al., 2019). The overall average of these values from an entire sequence of responses is the forward flow. The control group showed significantly greater forward flow (semantic distance travelled) than the patients: *t*(25) = 3.61, *p* = .001, *d* = 1.39.

#### Analysis 2: Fluency controlled creative potential

##### Uniqueness

Responses produced by only one participant were awarded one point for uniqueness with all other responses scored zero. The percentage of unique responses in relation to the number of non‐unique responses for each time window was then computed. Thus, the *relative* number of unique responses was calculated.

**FIGURE 6:**
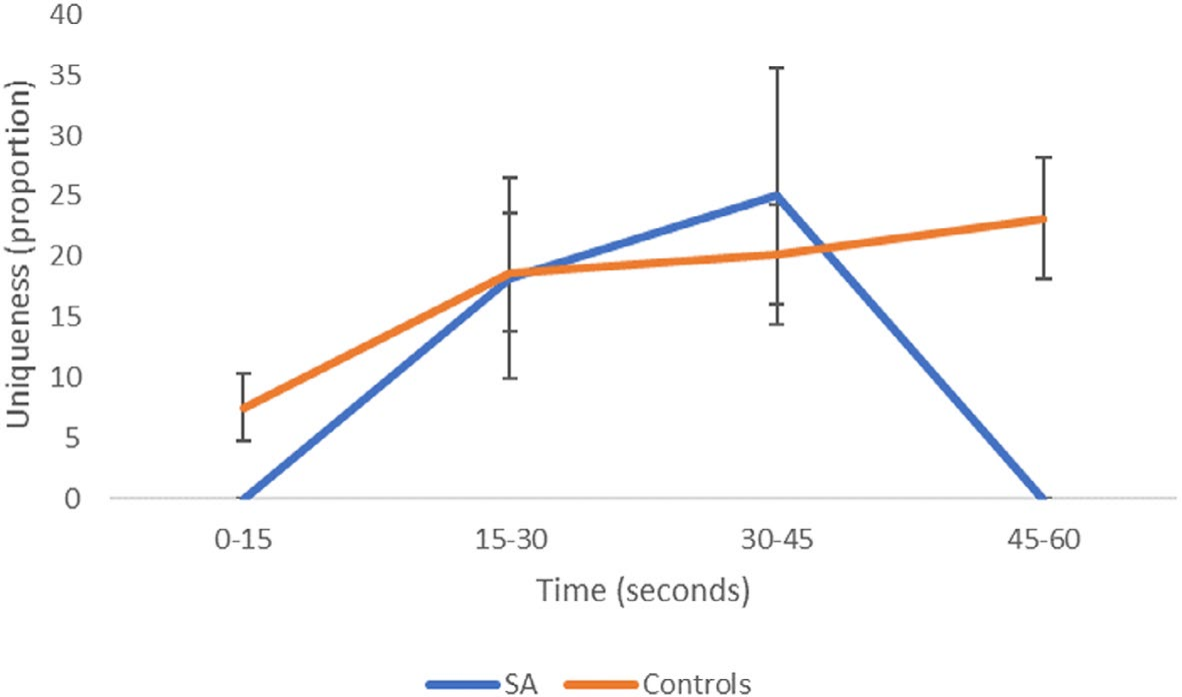
Percent unique responses produced across time for patients and controls. Error bars show standard error of the mean.

There was no group difference in uniqueness overall: *F*(1,25) = 1.582, *p* = .220, *η*_p_^2^ = .060, although Bayesian analysis indicated that models containing group were a little more likely (BF_incl_ = 1.14). However, the groups behaved differently across time, leading to an interaction of time and group: *F*(3,75) = 3.211, *p* = .028, *η*_p_^2^ = .114. Patients produced no unique responses at the first and last timepoints. The main effect of time was significant: *F*(3,75) = 5.907, *p* = .001, *η*_p_^2^ = .191, and there was a significant linear trend: *F*(1,25) = 6.883, *p* = .015, *η*_p_^2^ = .216. This is shown in Figure 6. An analysis of relative uniqueness showed a similar pattern of results and is displayed in Supplementary Analysis S5.

##### Creativity

Because producing more responses can lead to greater creativity, we next controlled for fluency by running an ANCOVA with fluency as a co‐variate. Forty raters who did not participate in the main study took part in rating creativity of responses. Each rater was asked to consider whether each answer to the category Animals was a ‘creative response’ using the same procedure as described for Experiment 2. Rater responses were removed if they did not utilise the full range of the scale, or they did not fully complete the questionnaire. That left 27 sets of responses [11 male, 16 female; M (SD): age = 31 (11.45), age left education = 21 (2.56)]. Rating scores can be seen in Table S4.

Average creativity per time window was calculated. There was a main effect of fluency on average creativity: *F*(1,24) = 7.388, *p* = .012, *η*_p_^2^ = .235. With fluency as a covariate, group effects were not significant: *F* < 1, BF_incl_ = .57, nor was time: *F*(3,72) = 1.994, *p* = .122, *η*_p_^2^ = .077. The interaction of time and fluency was not significant: *F*(3,72) = 1.678, *p* = .179, *η*_p_^2^ = .065. There was no interaction of time and group: *F* < 1, BF_incl_ = .46. This is shown in Figure S2.

An analysis of matched responses—matching each patient with a control based on their demographics and stopping analysis when patients had finished responding—was also run. There were no differences between groups. This is displayed in Supplementary Analysis S6.

### Interim discussion

The findings suggest that executive strategies such as exploiting a subcategory (cluster size) as well as exploring new subcategories (switching) can increase the volume of responses produced. However, despite the significantly reduced *quantity* of responses, uniqueness and creativity were similar between groups—when fluency was taken into account. This aligns with evidence from Experiment 2 that suggests patients can achieve creative potential through spreading activation, despite deficient semantic control mechanisms. However, it is likely that spreading activation alone is limited. There was a clear temporal impact of responses, and the majority of patients failed to respond during the final timepoint. This suggests that, for sustained fluency, executive mechanisms interact with spreading activation capabilities.

## OVERALL DISCUSSION

This study explored the impact of semantic control deficits on creative potential by manipulating task demands across category judgment and fluency tasks. Findings support the associative thinking model of creativity (Beaty & Kenett, 2023) by providing, for the first time, neuropsychological evidence on how spreading activation in the context of impaired semantic control can underpin creative potential.

For the fluency tasks, lack of semantic control increased the reliance on spreading activation for creative potential. This means strategies like switching and clustering were less common, limiting the total number of responses produced without impacting the creativity or uniqueness of responses. Patients were more likely to overstep a category boundary and produce an invalid response, but were also more likely to produce a unique response, potentially as a function of idiosyncratic, individual semantic networks. Patients particularly benefited from low coherence categories that gave more space for exploration, with a wide scope of valid responses. However, greater uniqueness did not necessarily result in greater judged creativity for SA patients, most likely because unique responses, while still being judged valid, may sometimes be considered less appropriate.

Patients showed greater inflexibility, with a stronger influence of what they had previously produced, cueing themselves through a category and occasionally out of it. This reduced flexibility may reflect a greater reliance on local relevance in the context of more difficulty with maintaining global coherence (Hoffman et al., 2020). It may be that patients have particular difficulty in evaluating the validity of their responses, a process that relies on prefrontal regions (Kleinmintz et al., 2019).

SA patients showed deficits in the category judgement task that were not seen in the category fluency tasks. Patients were unable to shape retrieval to fit a pre‐defined association, while inhibiting distractors, an impairment commonly seen in SA (Jefferies & Lambon Ralph, 2006; Noonan et al., 2010; Thompson et al., 2022). However, patients may be able to use internal networks in the *generation* of creative responses. Participants had great freedom in their responses—there were thousands of responses, and very few deemed invalid by raters. This freedom played to the patients' strengths—where there were limited task goals to keep in mind, and little need to shape responses to something pre‐defined.

There are a number of future avenues to explore. Firstly, the atypical and inappropriate responses were more common in SA. Patients are likely to have difficulty evaluating the appropriateness of responses (Chrysikou, 2019). This may lead to the generation of idiosyncratic or tangential responses that diverge from task demands. Importantly, these atypical responses may not be solely the loss of control mechanisms but could also reflect long‐standing disruptions within the semantic representational system itself. Prolonged periods of dysregulated semantic activation may result in an increasingly disorganised or distorted semantic network. Thus, the boundary between creative semantic processing and disordered retrieval may be blurred in SA, particularly when monitoring and feedback mechanisms are also compromised.

Second, we present data from one high coherence category, Animals, in the unconstrained experiment, but further exploration, including low coherence categories, may be informative. As time elapses, executive strategies are increasingly important to access less‐associated, more creative responses (Beaty & Silvia, 2012). Controls produced their most unique responses at the final timepoint, whereas patients produced none, potentially reflecting the limits of spreading activation. Low coherence categories cannot rely as heavily on executive strategies, such as clustering and switching, as they are less well defined. This may be beneficial to patients who are less able to use these mechanisms.

Thirdly, self‐generation of responses is rarely explored in SA (but see Hoffman et al., 2020). SA patients have been shown to have profound deficits in selecting targets amongst distractors (Noonan et al., 2010; Thompson et al., 2022). Patients are greatly impacted by task demands. Tasks that involve self‐generation place demands on the internal mechanisms of constraint, but performance may flourish when the task does not require a specific response. This may have consequences for research about the nature of semantic impairments, as well as having potential implications for therapy.

Finally, an important limitation is the reliance on human raters, most notably for the unrestricted fluency task which had mostly a single rater designate clusters. New approaches are emerging that can provide reliable and automated analyses of fluency data (Kumar et al., 2024; Zemla et al., 2020). Further work could incorporate these approaches.

## CONCLUSION

Overall, this study finds spreading activation, intact in patients, is sufficient to produce comparable responding to controls across several tasks tapping creative potential. Despite disrupted semantic control, as evident in the category judgment task, patients only show restrictions in their strategies to maintain high levels of fluency, rather than producing less creative or unique responses. The discrepancy in performance between responding to pre‐selected items and self‐generation is noteworthy and has important therapeutic and research implications.

## AUTHOR CONTRIBUTIONS

**Hannah E. Thompson:** Conceptualization; methodology; formal analysis; data curation; writing – original draft; visualization; supervision. **Paul T. Sowden:** Conceptualization; methodology; formal analysis; data curation; writing – original draft; visualization; supervision. **Lucy Cogdell‐Brooke:** Conceptualization; methodology; data curation; writing – review and editing; funding acquisition. **Ines R. Violante:** Methodology; software; formal analysis; visualization; supervision. **Beth Jefferies:** Data curation; writing – review and editing.

## FUNDING INFORMATION

This research was supported by the University of Surrey PhD studentship award to L.C.B.

## CONFLICT OF INTEREST STATEMENT

The authors declare no conflicts of interest.

## Supporting information


Appendix S1

Table S1

Table S3

## Data Availability

Public archiving of brain data was not possible due to consent limitations; therefore, researchers who wish to access these data should contact the corresponding author. All anonymised data, materials and code for the category fluency and category judgement tasks, including rating procedures and scoring instructions, are available on the Open Science Framework at: https://osf.io/nxa23/. Due to legal copyright restrictions, some neuropsychological tests used in background assessments (e.g. WMS‐IV, Brixton Spatial Anticipation Task) cannot be archived, but other materials are available in the repository.
