# Supplementary material for: Category fluency and creative potential in semantic aphasia
Source: J Neuropsychol. 2025 Dec 10;20(1):154–74. doi: 10.1111/jnp.70019 (PMC12976839; doi:10.1111/jnp.70019)
Supplement: Supplementary file 1 — Appendix S1 [file JNP-20-154-s001.docx]

**Supplementary materials**

Supplementary Figure S1: Average creativity scores in category fluency for SA and controls. Error bars show standard error of mean.

.

Supplementary Figure S2: Creativity scores with the covariate fluency across time for patients and controls. Covariates appearing in the model are evaluated at fluency = 17.3. Errors bars show the standard error of mean.

Supplementary Table S1*:* See Excel spreadsheet

Supplementary Table S2*:* Convergent task categories, targets and distractors

| **Dimension** | **Category** | **Target** | **Categorical** | **Associated** | **Unrelated** |
| --- | --- | --- | --- | --- | --- |
| High | Farm animals | Chicken, cow, horse, pig, sheep. | Crocodile, tiger, camel, kangaroo, lobster. | Well, tree, corn, barn, axe. | Boat, star, cymbal, pineapple, ear. |
| High | Items of clothing on top half | Jumper, dress, blouse, shirt, tie | Shoe, skirt, handbag, ring, hat | Hairbrush, hair, iron, draws, hanger. | Potato, pencil, bread, violin, zebra. |
| High | Objects that hold water | Glass, kettle, vase, watering can, mug. | Well, fridge, water drop, snowman, cloud. | Lobster, fish, seahorse, crocodile, mermaid. | Motorcycle, squirrel, bed, lemon, pram. |
| High | Vegetables. | Pepper, carrot, lettuce, onion, pumpkin. | Apple, banana, pear, cherry, melon. | Grass, flower, plate, fork, knife. | Lock, penguin, bike, guitar, gorilla. |
| High | Transport. | Plane, bus, car, helicopter, train. | Traffic light, road, sign, sign, track. | Key, suitcase, bridge, stop, helmet. | Trumpet, pig, asparagus, fox, dress. |
| High | Exotic fruits. | Pineapple, passionfruit, papaya, kiwi, mango. | Apple, cherry, pear, banana, grapes. | Palm tree, beach, sun, hammock, and umbrella. | Bowl, dustbin, dress, bear, fork. |
| High | Facial features. | Nose, eye, eyebrow, ear, lips. | Arm, hair, thumb, foot, hand. | Glasses, necklace, lipstick, brush, toothbrush. | Carrot, cow, kangaroo, salt, kettle. |
| High | Insects. | Ant, caterpillar, beetle, grasshopper, fly. | Racoon, ostrich, horse, tortoise, tiger. | Leaf, grass, tree, apple, lettuce. | Chair, rolling pin, harp, needle, kite. |
| High | Animals that live in water. | Seal, crab, fish, seahorse, shark. | Cat, camel, cow, elephant, giraffe. | Boat, wave, drop, shell, seaweed. | Skirt, ring, tomato, ear, lemon. |
| High | Kitchen appliances. | Kettle, pan, frying pan, cooker, fridge. | TV, desk, radio, bed, dressers. | Salt, cake, bread, cheese, house. | Cloud, dog, key, rhino, cactus. |
| High | Dangerous animals. | Gorilla, crocodile, rhino, lion, tiger. | Seahorse, penguin, snail, squirrel, caterpillar. | Razor, gun, chain, fangs, axe. | Snowman, onion, watermelon, truck, tie. |
| High | Flying animals. | Owl, eagle, fly, bat, butterfly. | Racoon, skunk, rabbit, lobster, sheep. | Cloud, helicopter, aeroplane, tree, feather. | Lock, toaster, potato, chair, hands. |
| High | Zoo animals. | Tiger, zebra, gorilla, elephant, penguins. | Fly, bee, ant, duck, pig. | Tent, cage, fence, palm tree, tree. | Pumpkin, barn, piano, traffic light, can. |
|  |  |  |  |  |  |
| Low | Things that are round. | Ball, moon, sun, ring, orange. |  | Cloud, racket, bat, banana, bowl. | Monkey, finger, tree, church, pliers, snake, player, nose, mountain, strawberry. |
| Low | Made of wood. | Barrel, desk, ladder, fence, bat. |  | Basket, tree, saw, hammer, screwdriver. | Lettuce lips, cymbals, gorilla, racoon, ostrich, pumpkin, star, thumb. |
| Low | Small. | Needle, button, ant, nut, acorn. |  | Star, moon, chick, baby, lamb. | Iron, jug, necklace, lobster, flute, rhino, trumpet, lamp, frog, chisel. |
| Low | Large. | Plane, moon, mountain, star, sun. |  | rhino, house, barn, elephant, truck. | Celery, accordion, anchor, asparagus, bowl, sofa, drum, cow, football, eye. |
| Low | Soft. | Feather, wool, towel, bunny, cat. |  | Hair, bread, lips, harp, music. | Cake, bed, cheese, cloud, brush, fork, asparagus, pig, waistcoat, oven. |
| Low | Handles. | Gun, kettle, fork, axe, toothbrush. |  | Glass, sofa, bed, house, bowl. | Fox, wheel, jumper, snail, peanut, skates, hair, potato, swan, thimble. |
| Low | Thin. | Feather, needle, leaf, envelope, thread. |  | Flute, knife, asparagus, pencil, chain. | Pumpkin, pineapple, heart, bin, thumb, crown, mushroom, vase, doll, trousers. |
| Low | Stripes. | Bee, football, zebra, ruler, tiger. |  | Giraffe, harp, lion, paintbrush, comb. | Lock, table, eye, pear, violin, window, scissors, coach, lips, dress. |
| Low | Make a noise | Chicken, lips, flute, gun, record player |  | Note, ant, fish, nose, had | Grapes, foot, hanger, lemon, needle, ruler, vase, window, shirt, pineapple. |
| Low | Square | Suitcase, window, book, envelope, TV |  | House, handbag, car, ruler, accordion | Leaf, kangaroo, hair, key, finger, scissors, mountain, orange, moon, lobster. |
| Low | Move on wheels | Cart, train, car, skates, pram. |  | Plane, yacht, helicopter, wheel, sledge. | Plug, kettle, rhino, lock, plum, harp, gorilla, dog, fork, coat. |

Supplementary Table S3*:* See Excel spreadsheet

Supplementary Table S4: Creativity scores calculated from scores given by independent raters.

| Animal | Average Score | Std. Deviation |
| --- | --- | --- |
| Dog | 0.89 | 1.58 |
| Cat | 0.89 | 1.37 |
| Mouse | 0.96 | 0.81 |
| Rat | 1.00 | 0.78 |
| Fish | 1.00 | 1.06 |
| Horse | 1.15 | 1.35 |
| Cow | 1.15 | 1.51 |
| Sheep | 1.19 | 1.11 |
| Chicken | 1.19 | 1.00 |
| Pig | 1.22 | 1.37 |
| Rabbit | 1.26 | 1.43 |
| Snake | 1.26 | 1.16 |
| Bird | 1.35 | 1.85 |
| Duck | 1.44 | 1.09 |
| Goat | 1.52 | 0.85 |
| Hamster | 1.56 | 0.97 |
| Lamb | 1.56 | 0.97 |
| Monkey | 1.59 | 1.62 |
| Lion | 1.59 | 1.39 |
| Hen | 1.67 | 0.78 |
| Goose | 1.70 | 1.44 |
| Squirrel | 1.74 | 1.79 |
| Donkey | 1.81 | 1.33 |
| Pony | 1.85 | 1.35 |
| Elephant | 1.85 | 1.70 |
| Frog | 1.89 | 1.40 |
| Lizard | 1.89 | 1.45 |
| Fox | 1.93 | 1.30 |
| Guinea Pig | 1.93 | 1.62 |
| Spider | 1.96 | 1.81 |
| Fly | 2.00 | 1.67 |
| Turkey | 2.00 | 1.20 |
| Tortoise | 2.04 | 1.32 |
| Parrot | 2.04 | 1.58 |
| Turtle | 2.04 | 1.51 |
| Bear | 2.07 | 1.47 |
| Bull | 2.07 | 1.38 |
| Snail | 2.15 | 1.43 |
| Seagull | 2.19 | 1.20 |
| Swan | 2.19 | 1.49 |
| Ape | 2.22 | 1.58 |
| Owl | 2.22 | 1.25 |
| Crocodile | 2.26 | 1.35 |
| Bee | 2.27 | 1.69 |
| Gorilla | 2.30 | 1.30 |
| Ant | 2.32 | 1.60 |
| Wasp | 2.32 | 1.46 |
| Robin | 2.33 | 1.36 |
| Greyhound | 2.37 | 1.15 |
| Shark | 2.37 | 1.36 |
| Tiger | 2.41 | 1.50 |
| Butterfly | 2.50 | 1.66 |
| Giraffe | 2.52 | 1.60 |
| Zebra | 2.52 | 1.53 |
| Seal | 2.52 | 1.45 |
| Blackbird | 2.56 | 1.65 |
| Wolf | 2.59 | 1.31 |
| Eagle | 2.59 | 1.19 |
| Chimp | 2.63 | 1.60 |
| Dolphin | 2.63 | 1.60 |
| Sparrow | 2.67 | 1.27 |
| Penguin | 2.67 | 1.82 |
| Moth | 2.68 | 1.44 |
| Grasshopper | 2.72 | 1.21 |
| Deer | 2.74 | 1.87 |
| Cheetah | 2.74 | 1.61 |
| Bat | 2.78 | 1.48 |
| Whale | 2.81 | 1.55 |
| Hedgehog | 2.81 | 1.71 |
| Badger | 2.85 | 1.49 |
| Budgie | 2.85 | 1.64 |
| Cricket | 2.88 | 1.54 |
| Rooster | 2.89 | 1.67 |
| Hippo | 2.89 | 1.58 |
| Kangaroo | 2.89 | 1.34 |
| Collie | 2.89 | 1.85 |
| Panda | 2.89 | 1.65 |
| Otter | 2.89 | 1.42 |
| Gerbil | 2.89 | 1.78 |
| Worm | 2.89 | 1.83 |
| Woodpecker | 2.93 | 1.64 |
| Racoon | 2.93 | 1.69 |
| Hawk | 3.00 | 1.57 |
| Leopard | 3.04 | 1.43 |
| Hare | 3.11 | 1.63 |
| Alligator | 3.15 | 1.54 |
| Cod | 3.15 | 1.83 |
| Boar | 3.15 | 1.49 |
| Ram | 3.15 | 1.66 |
| Tarantula | 3.15 | 1.62 |
| Ox | 3.19 | 1.73 |
| Jaguar | 3.19 | 1.42 |
| Bluebird | 3.19 | 1.71 |
| Cobra | 3.22 | 1.55 |
| Tuna | 3.26 | 1.63 |
| Orangutan | 3.26 | 1.46 |
| Peacock | 3.26 | 1.63 |
| Skunk | 3.30 | 1.75 |
| Rhino | 3.33 | 1.52 |
| Chipmunk | 3.33 | 1.64 |
| Ostrich | 3.33 | 1.39 |
| Llama | 3.33 | 1.49 |
| Ferret | 3.33 | 1.54 |
| Meerkat | 3.37 | 1.24 |
| Antelope | 3.37 | 1.50 |
| Blue Whale | 3.37 | 1.69 |
| Pheasant | 3.44 | 1.55 |
| Toad | 3.48 | 1.78 |
| Salmon | 3.48 | 1.78 |
| Panther | 3.48 | 1.31 |
| Falcon | 3.48 | 1.25 |
| Python | 3.52 | 1.45 |
| Finch | 3.56 | 1.60 |
| Polar Bear | 3.59 | 1.42 |
| Buffalo | 3.63 | 1.11 |
| Hummingbird | 3.63 | 1.28 |
| Kingfisher | 3.63 | 1.90 |
| Koala | 3.63 | 1.74 |
| Thrush | 3.73 | 1.71 |
| Weasel | 3.78 | 1.25 |
| Walrus | 3.85 | 1.66 |
| Stick Insect | 3.88 | 1.64 |
| Shrew | 3.89 | 1.65 |
| Hyena | 3.89 | 1.50 |
| Eel | 3.96 | 1.40 |
| Alpaca | 3.96 | 1.56 |
| Iguana | 4.00 | 1.33 |
| Cougar | 4.00 | 1.69 |
| Camel | 4.07 | 1.73 |
| Sealion | 4.07 | 1.66 |
| Sardine | 4.07 | 1.94 |
| Cockatoo | 4.07 | 1.41 |
| Coyote | 4.11 | 1.69 |
| Chinchilla | 4.15 | 1.56 |
| Anteater | 4.15 | 1.66 |
| Wallaby | 4.19 | 1.84 |
| Puma | 4.22 | 1.53 |
| Dinosaur | 4.28 | 2.23 |
| Mountain Lion | 4.30 | 1.54 |
| Chameleon | 4.30 | 1.77 |
| Gecko | 4.37 | 1.50 |
| Orca | 4.37 | 1.60 |
| Seabream | 4.38 | 1.98 |
| Mongoose | 4.41 | 1.82 |
| Herring | 4.41 | 1.80 |
| Sterling | 4.45 | 1.79 |
| Water Buffalo | 4.48 | 1.37 |
| Possum | 4.56 | 1.83 |
| Emu | 4.58 | 1.36 |
| Wildebeest | 4.62 | 1.44 |
| Porpoise | 4.63 | 1.42 |
| Dingo | 4.78 | 1.72 |
| Salamander | 4.78 | 1.83 |
| Tapeworm | 4.84 | 1.95 |
| Aardvark | 4.96 | 1.37 |
| Platypus | 5.00 | 1.75 |
| Bush Baby | 5.12 | 1.93 |
| Lynx | 5.15 | 1.32 |
| Dixon | 5.28 | 1.64 |
| Mink | 5.44 | 1.50 |
| Dragon | 5.47 | 1.68 |
| Marmoset | 5.52 | 1.78 |
| Oryx | 5.56 | 1.55 |
| Civet | 5.62 | 1.50 |
| Okapi | 5.81 | 1.75 |
| Coypu | 6.00 | 1.44 |
| Sidewinder | 6.04 | 1.37 |

Average scores indicate mean creativity scores (0-7).

**Supplementary Analysis S1: Analysis of neuroimaging data**

In order to identify which areas were affected in the participants, we conducted a lesion analysis to produce a map of damage in the sample. We manually defined abnormal brain tissue using T1, T2 and FLAIR MRI images for each available patient that met the scanning criteria (Fiez, Damasio, & Grabowski, 2000; Seghier, Ramlackhansingh, Crinion, Leff, & Price, 2008). We aligned each modality in subject space and performed lesion tracing using an image segmentation tool developed in collaboration with Imperial College London (ImSeg). To ensure lesion tracing was accurate, brain tissue was additionally visually compared between patients and controls. T1 images from each patient were pre-processed using the FMRI Expert Analysis tool (FEAT Version 6.00) from the FMRIB Software Library (FSL; Jenkinson, Beckmann, Behrens, Woolrich, & Smith, 2012; Smith et al., 2004). We performed brain extraction to remove non-brain tissue (BET; Smith, 2002) and manually adjusted where necessary. This was followed by normalising T1 and brain lesion masks to Montreal Neurological Institute (MNI) 152 standard space using FMRIB Linear Image Registration tool (FLIRT). All patients had damage to one or more areas of the semantic control network (Jackson, 2021; Noonan et al., 2013). Lesions centred within the left temporal lobe, with severe damage in six out of seven patients to an area within the IFG (BA 44 & 45), and partial damage in four patients across the middle and pre-central gyrus (BA 9, 10, 46, 4 & 6). The insula was damaged in all patients (BA 13). Three patients also exhibited partial damage to the left IPL (BA 40, 39) and two to the posterior MTG (BA 21).

**Supplementary Analysis S2: Errors in category judgement**

We explored error types across groups. Overall, patients were more likely to make errors which were further removed from the target, leading to an interaction of error type and group: F(3,87) = 17.021, p < .001, η_p_^2^ = .378. For high coherence categories, there was a main effect of error type: F(3,84) = 319.789, p < .001, η_p_^2^ = .919. No other effects were significant: F < 1. When looking at each error type (averaged across modalities), controls produce more categorical errors than SA: t(33) = 2.431, p = .021, *d* = .885, and SA produce more associative errors than controls: t(33) = 2.342, p = .025, *d* = .853. There were no other significant differences (t ≤ 1.449, p ≥ .178). This is shown in Supplementary Figure S3.

Supplementary Figure S3: Proportion of error type for patients and controls for high coherence category judgement

For low coherence categories, there was a main effect of error type: F(2,56) = 430.145, p < .001, η_p_^2^ = .939, and interaction with group: F(2,56) = 10.970, p < .001, η_p_^2^ = .281. No other significant effects were found: F ≤ 1.694, p ≥ .193. Averaging across modality, there was a group difference for semantic errors, with controls producing more related errors: t(33) = 5.143, p < .001. No other group effects reached significance (t(33) ≤ 1.814, p ≥ .094). This is shown in Supplementary Figure S4.

Supplementary Figure S4: Proportion of error type for patients and controls for low coherence category judgement

**Supplementary Analysis S3: Invalid responses**

As part of scoring items for creativity (see section 4.2.4), 45 raters, who did not participate in the main study [15 male, 30 females; M (SD) Age = 38 (16.47); Age left education = 21 (2.56)], were split into two groups and randomly assigned 12 of the 24 categories each, so each item was rated by at least 22 raters. When two or more raters flagged an item as invalid, this was excluded from analysis after checking by an experimenter.

For high coherence, of 3832 responses, 41 were invalid (1.1%). For low coherence, of 3167 responses, 24 were invalid (0.8%). There were 2 repetition errors. As there were so few invalid responses, responses across modalities were combined.

There was a significant group difference, with SA cases producing more invalid responses: F(1,34) = 7.630, p = .009, η_p_^2^ = .183. There was a significant difference between high and low coherence, with more invalid responses for high coherence: F(1,34) = 4.962, p = .033, η_p_^2^ = .127. There was also an interaction, with SA patients producing more invalid responses in the high coherence category: F(1,34) = 5.973, p = .020, η_p_^2^ = .149. There was a significant group difference for high coherence: t(34) = 2.775, p = .009, *d* = 1.004, but not low coherence: t(34) = 1.226, p = .229, *d* = .443. This is shown in Supplementary Figure S5.

Supplementary Figure S5: Invalid responses produced by SA and controls according to category type. Error bars show standard error of mean.


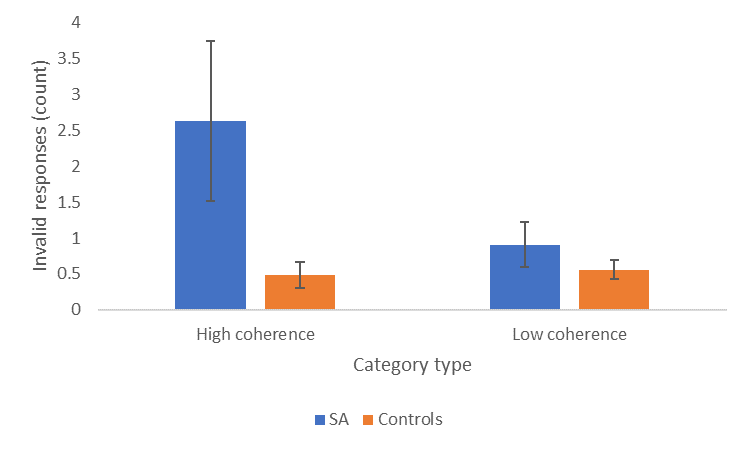


**Supplementary Analysis S4: Association between category prompt and responses**

In an analysis of the similarity of responses to the category prompt, there was a significant main effect for category coherence: F(1,20) = 1065.60, *p* < .001, η_p_^2^ =.982. This reflects higher similarity levels between responses and the category prompt in the high compared to low coherence categories. No other effects reached significance: F ≤ 2.763, p ≥ .112. This is shown in Supplementary Figure S6.

Supplementary Figure S6. Average similarity scores (max. possible = 1) between category prompt and responses for each modality and level of category coherence for patients and controls. Error bars show standard error of the mean.

**Supplementary Analysis S5: Relative uniqueness**

The relative uniqueness was calculated by dividing the number of times a given response was produced across the whole sample by the total sample size (27) and subtracting the result from 1. A response given by the whole sample would therefore have a uniqueness score of 0, whereas a response produced by just one member of the sample would have a uniqueness score of 1-(1/27) = .963. Relative uniqueness was then averaged across responses for each time point.

Eight of the patients and one control made no responses in one or two of the time windows. These missing values were replaced by calculating the respective group mean for that time point and then weighting it by the individual participant’s mean uniqueness score across all their responses, relative to the maximum mean from participants in the same group.

For relative uniqueness, there was no effect of fluency: F(1,24) = 3.424, p = .077, η_p_^2^ = .125. Time was significant: F(3,72) = 4.566, p = .006, η_p_^2^ = .160, but group was not: F < 1. The linear assumption held for time: F(1,24) = 11.990, p = .003, η_p_^2^ = .333.

Time interacted with fluency: F(3,72) = 3.100, p = .032, η_p_^2^ = .114. The linear assumption held for this interaction: F(1,24) = 18.255, p < .001, η_p_^2^ = .432. There was no interaction of time and group: F(3,72) = 1.819, p = .151, η_p_^2^ = .070. This is shown in Supplementary Figure S7.

Supplementary Figure S7: Relative uniqueness with fluency as a covariate for patients and controls

Covariate appearing in the model as: 17.3. Error bars show standard error of mean.

**Supplementary Analysis S6: Matched responses**

Each patient was matched with a control based upon their age, gender and educational history (t < 1). As the numbers were slightly uneven, this removed one control (C7). Originally, patients produce an average of 8 responses, and controls 26 responses. We therefore stopped analysing control responses when patients had finished responding for each matched pair. Descriptive statistics are presented in Supplementary Table S5.

Supplementary Table S5: Descriptive statistics on a matched analysis of SA and control participants

|  | SA | Controls |
| --- | --- | --- |
| Total responses | 7.8 (4.1) | Matched |
| Total switches | 3 (2.3) | 2.8 (2.7) |
| Average cluster size | 2.1 (0.6) | 2.3 (0.7) |
| Total creativity | 15.8 (9.5) | 16.5 (10.3) |
| Unique responses | 0.8 (1.1) | 0.6 (1.1) |
| Relative uniqueness | 0.6 (0.1) | 0.6 (0.1) |

There were no significant differences in the number of switches (t < 1). There were no differences in cluster size: t(24) = 1.041, p = .308, *d* = .408.

There was no difference in relative uniqueness, total creativity, or total unique responses (t < 1). Interestingly, there was a total of 72 unique responses, of which only 19 were captured in this matched pairs analysis (meaning 53/72, or 74%, came from controls after patients had stopped responding).
